# Heparin improves the mortality of patients with non-pulmonary sepsis-associated ARDS: A MIMIC-IV database analysis based on propensity score matching

**DOI:** 10.1371/journal.pone.0333795

**Published:** 2025-10-10

**Authors:** Jinfeng Lin, Zhilong Cao, Chunfeng Gu, Lijun Tian, Yadong Wang, Xudong Han

**Affiliations:** 1 Critical Care Medicine, Nantong Third People`s Hospital, Affiliated Nantong Hospital 3 of Nantong University, Nantong, China; 2 Ctrip Infrastructure Service, Trip.com Group Ltd, Shanghai, China; Azienda Ospedaliero Universitaria Careggi, ITALY

## Abstract

**Background:**

Non-pulmonary sepsis often induces Acute Respiratory Distress Syndrome (ARDS). Dysregulated inflammation and coagulation disorders play important roles in the development of non-pulmonary sepsis-associated ARDS (NPS-ARDS). Heparin, with its potential anticoagulant and anti-inflammatory properties, may be used in the treatment of NPS-ARDS.

**Methods:**

This is a retrospective observational study that uses Structured Query Language (SQL) to extract clinical data of NPS-ARDS patients from the Medical Information Mart for Intensive Care (MIMIC)-IV database. Based on the dosage of heparin, patients were divided into three groups: low-dose heparin treatment group (0-5000u/d), medium-dose heparin treatment group (5000u-10000u/d), and high-dose heparin treatment group (greater than 10000u/d). Propensity score matching (1:1) was used to match similar patients from the NPS-ARDS patients who did not use heparin to each heparin treatment group. The study compares the effects of heparin at different dosages on short-term mortality (7-day, 28-day, and 60-day mortality) and one-year cumulative survival rate in NPS-ARDS patients.

**Results:**

PSM reduced the impact of confounding factors on the results to some extent. Low and medium doses of heparin did not improve patient mortality. However, high-dose heparin improved the short-term mortality of NPS-ARDS patients (7-day mortality: 4.1% vs. 14.3%, P < 0.001; 28-day mortality: 9.4% vs. 22.6%, P < 0.001; 60-day mortality: 13.2% vs. 24.8%, P = 0.001) and one-year cumulative survival rate (Log Rank = 8.349, P = 0.004), but it also prolonged ICU stay (6.7 ± 6.2 days vs. 5.7 ± 4.8 days, P = 0.041) and invasive mechanical ventilation (11.7 ± 6.9 hours/day vs. 5.7 ± 4.8 hours/day, P < 0.001).

**Conclusion:**

In patients with NPS-ARDS, high-dose heparin was associated with significantly improved short- and long-term survival, albeit at the cost of prolonged ICU stay and mechanical ventilation.

## Introduction

Acute Respiratory Distress Syndrome (ARDS) is a common condition in intensive care units, characterized by acute respiratory failure due to diffuse inflammation and edema in the lungs [1]. Patients with ARDS in the ICU often have poorer outcomes [2]. Pulmonary infections, sepsis, aspiration, trauma, pancreatitis, transfusion, and drowning can lead to ARDS, with sepsis being a significant cause of ARDS [3]. In clinical practice, physicians often focus on ARDS caused by pulmonary infections while overlooking ARDS resulting from non-pulmonary infections (such as bloodstream infections, abdominal infections, and urinary tract infections). This oversight may delay the diagnosis and treatment of these patients [4]. Coagulation disorders and dysregulated inflammatory responses play important roles in the development and progression of ARDS. In ARDS, there is an enhancement of pro-coagulation and reduced fibrinolysis within the alveoli, leading to the formation of microthrombi and accumulation of fibrin. This impairs alveolar gas exchange, resulting in persistent hypoxemia in patients [5]. Heparin is a commonly used anticoagulant that also has anti-inflammatory properties. It can reduce the deposition of fibrin and decrease the formation of microthrombi in the alveoli, thereby potentially alleviating ARDS to some extent [6]. Current research mainly focuses on the use of heparin for treating sepsis or ARDS [7]. There is limited research on the use of heparin for treating ARDS caused by sepsis, especially regarding the treatment of non-pulmonary sepsis-associated ARDS (NPS-ARDS). In preliminary studies, our team developed a machine learning model to predict the occurrence of ARDS in patients with non-pulmonary sepsis [8]. When dealing with non-pulmonary infection sources of sepsis patients, we can accurately predict the likelihood of ARDS in these patients. To enable early treatment for such high-risk patients, this study uses the MIMIC database to investigate the impact of heparin on mortality rates in NPS-ARDS patients, aiming to provide evidence for the use of heparin in non-pulmonary sepsis patients at high risk of developing ARDS.

## Methods and materials

### Data source

The data for this study comes from the MIMIC database, which contains information on critically ill patients admitted to the Beth Israel Deaconess Medical Center (BIDMC) between 2008 and 2019. Our analysis included patient data from this entire period (2008–2019). The MIMIC database is a large clinical database jointly developed by BIDMC and the Massachusetts Institute of Technology (MIT) [9,10]. The MIMIC database provides a wealth of research data to scholars worldwide. The author of this paper, Jinfeng Lin, has completed the CITI training program for MIMIC database collaborators and holds a license (number: 48886665), granting access to the MIMIC database. The data were accessed for research purposes from May 1, 2024 to May 30, 2024. The authors did not have access to information that could identify individual participants during or after data collection.

### Ethics approval and consent to participate

All methods were carried out in accordance with relevant guidelines and regulations. All the experimental protocols were approved by the Institutional Review Boards (IRBs) of Beth Israel Deaconess Medical Center and the Massachusetts Institute of Technology. A waiver of informed consent for the use of MIMIC‑IV data was given by the Institutional Review Boards (IRBs) of Beth Israel Deaconess Medical Center and the Massachusetts Institute of Technology.

### Patient and public involvement

This study did not include minors. No patients or members of the public were involved in the design, conduct, reporting, or dissemination of this research. This determination aligns with China’s Ethical Guidelines for Secondary Use of Health Data (2021 Edition) as the study exclusively analyzed pre-existing anonymized records without direct patient contact.

### Inclusion criteria

①Meets the Sepsis-3.0 diagnostic criteria [11]: presence of infection and a Sequential Organ Failure Assessment (SOFA) score ≥ 2; ②Requirement for mechanical ventilation (invasive mechanical ventilation and/or high-flow oxygen therapy) or an oxygenation index < 300 [12].

Define “presence of infection” as the use of antibiotics in combination with microbiological specimen collection, specifically: microbiological specimens were collected within 24 hours after the initiation of antibiotic therapy, or antibiotics were administered within 72 hours after specimen collection.

### Exclusion criteria

①Age < 18 years or > 89 years; ②ICU stay of less than 48 hours; ③Pulmonary infection: Positive or suspected positive microbial culture of respiratory specimens (sputum, bronchoalveolar lavage, etc.); ④History of pulmonary disease (chronic obstructive pulmonary disease, chronic pulmonary heart disease, asthma, bronchiectasis, pneumoconiosis, etc.); ⑤Use of heparin for blood purification; ⑥Use of other anticoagulants such as enoxaparin or warfarin.

### Experimental procedure

#### Baseline characteristics.

The baseline parameters for each group include general patient information (gender, age, Charlson Comorbidity Index), mean values of vital signs(heart rate, body temperature, respiratory rate, mean arterial pressure) during the first 24 hours in the ICU, and mean values of laboratory tests(arterial blood gas analysis, complete blood count, liver function, kidney function, and coagulation profile) from 6 hours before ICU admission to 24 hours within the ICU. These baseline parameters will also be used as the parameters for propensity score matching (PSM).

#### Prognosis.

The primary outcomes are short-term mortality (7-day, 28-day, and 60-day mortality) and long-term survival rates (one-year cumulative survival rate), while the secondary outcomes are ICU length of stay and the duration of various respiratory support treatments (invasive mechanical ventilation, non-invasive mechanical ventilation, oxygen therapy, high-flow oxygen therapy). The respiratory support treatment time (h/day) is calculated as the total duration of each respiratory support treatment divided by the total number of ICU days.

#### Procedure.

Using Structured Query Language (SQL), extract clinical data of NPS-ARDS patients from the MIMIC-IV database based on the inclusion and exclusion criteria. Retain patients with baseline data completeness greater than 95%, and perform multiple imputation for missing data.

**Fig 1:**
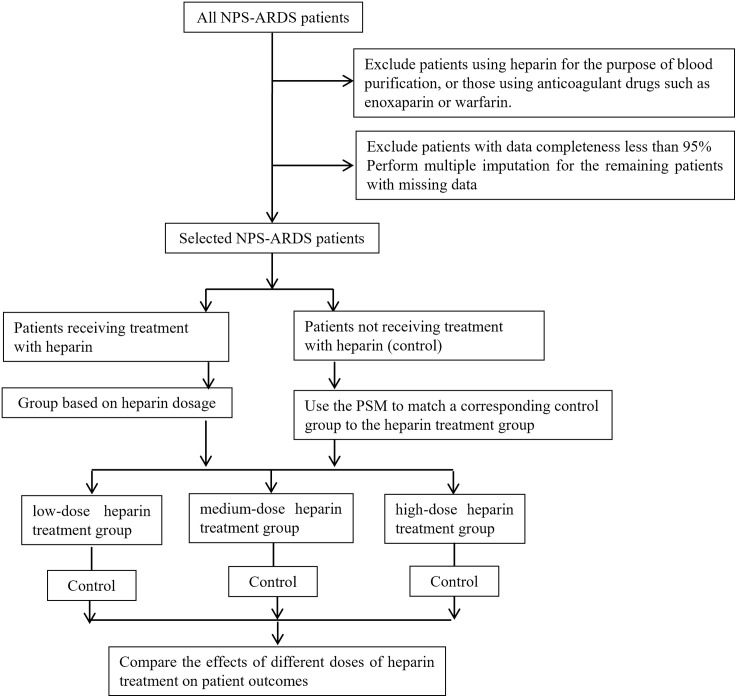
Experimental Flowchart. Divide patients into different heparin groups based on the amount of heparin used. Use PSM (1:1) to match similar patients from the NPS-ARDS patients who did not receive heparin with each heparin group. Compare the effects of different doses of heparin on the prognosis of NPS-ARDS patients.

All heparin administrations in this study were delivered subcutaneously. Based on the amount of heparin used, divide the heparin group patients into low-dose heparin group (0-5000u/d), medium-dose heparin group (5000u-10000u/d), and high-dose heparin group (greater than 10000u/d). Heparin dosage is calculated as the total amount of heparin used during the ICU stay divided by the total number of ICU days. The dose definition is a surrogate measure, not a precise pharmacological exposure metric. The dose groupings were pragmatically derived from standard prophylactic practices and the observed data distribution, rather than directly from prior ARDS-specific trials. Use propensity score matching (PSM, 1:1) to match similar patients from the NPS-ARDS patients who did not receive heparin with each heparin group.Compare the effects of different doses of heparin on the prognosis of NPS-ARDS patients. The specific procedure is shown in Fig 1.

Navicat Premium (version 15, PremiumSoft CyberTech Ltd) was used for data extraction and management. It provided a user-friendly interface for writing SQL queries, retrieving complex linked tables from the MIMIC-IV PostgreSQL database, and exporting structured datasets into formats suitable for further analysis. Navicat was chosen over other tools because it enables efficient data handling with reduced risk of manual conversion errors. Python (version 3.8, Python Software Foundation) was the primary tool for data preprocessing, multiple imputation, and propensity score matching (PSM). We used the pandas library for data cleaning and transformation. Python was chosen for these tasks due to its strong ecosystem for reproducible, script-based data processing and statistical modeling. After PSM was completed in Python, the matched datasets were imported into SPSS (version 25, IBM Corp.) for descriptive statistics, group comparisons, and survival analysis. In particular, SPSS was used to generate Kaplan–Meier survival curves and perform log-rank tests for cumulative survival rate comparisons. SPSS was chosen because it is widely used in clinical research for hypothesis testing and survival analysis, making results more interpretable to a broad medical readership.

#### PSM procedure.

(a) Load and process data: Use Pandas (an open-source data processing library in Python with functions for data import/export, cleaning, transformation, and aggregation) to read data from an Excel file and perform data cleaning (retain patients with ≥95% data completeness and impute missing values); (b) Build logistic regression model and estimate propensity scores: Use the logistic regression model from Scikit-learn (a Python module offering functions for regression, classification, clustering, and dimensionality reduction) to estimate each patient’s propensity score based on their baseline characteristics (X), and append the computed scores to the original dataframe; (c) Perform propensity score matching: Use the NearestNeighbors model to match each patient in the heparin treatment group with the most similar patient (in terms of propensity score) from the NPS-ARDS patients who did not receive heparin, thereby forming a control group; (d) Create and save matched dataset: Combine the heparin treatment group with their matched controls to generate a new matched dataset.

#### Statistical methods.

Continuous variable data are presented as mean ± standard deviation. Levene’s test for equality of variances is conducted for continuous variables. When variances are equal, independent t-tests are used to compare differences between two groups. When variances are not equal, the Mann-Whitney U test is used. For comparing the incidence rates between two groups, chi-square tests (χ² test), continuity-corrected chi-square tests, or Fisher’s exact tests are used.

For missing data, multiple imputation is used to fill in the gaps. Propensity scores are estimated using a logistic regression model, and matching is performed using the Nearest Neighbors model. The Standardized Mean Difference (SMD) is used to measure the difference in means between two groups for continuous data. Kaplan-Meier survival curves are used to compare the one-year cumulative survival rates between the two groups.

## Results

### Patients

In the MIMIC IV database, there were 7,632 patients who developed NPS-ARDS. Among them, 2,115 patients used heparin for the purpose of blood purification, or used other anticoagulants such as enoxaparin and warfarin during hospitalization.

**Fig 2:**
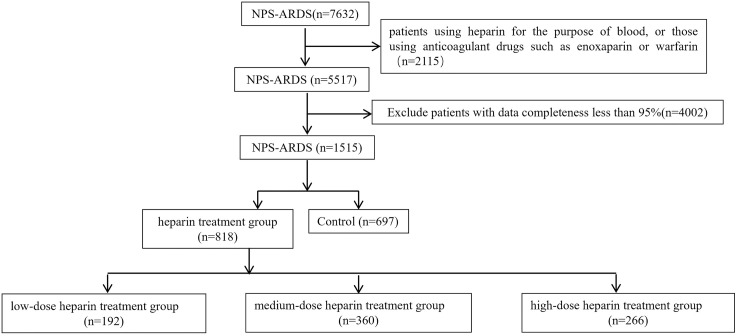
Flowchart of Patient Selection. 1,515 patients were included in this study, with 818 patients receiving heparin treatment and 697 NPS-ARDS patients who did not receive heparin.

Among the remaining 5,517 patients, 4,002 had data completeness less than 95%. Ultimately, 1,515 patients were included in this study, with 818 NPS-ARDS patients receiving heparin treatment. The low-dose heparin group included 192 patients, the medium-dose heparin group included 360 patients, and the high-dose heparin group included 266 patients. There were 697 NPS-ARDS patients who did not receive heparin. Details are shown in Fig 2.

### The effect of heparin use on the prognosis of NPS-ARDS patients

#### Baseline characteristics of the heparin use group and the control group (No heparin use).

This study included a total of 1,515 patients, with 818 patients in the heparin group and 697 patients in the control group. During propensity score matching (PSM), the control group was resampled to match 818 patients with those in the heparin group. Both groups primarily consisted of elderly patients. Compared to pre-PSM, the standardized mean differences (SMD) were significantly reduced after matching. Specifically, after PSM, the differences in platelet count, alanine aminotransferase, total bilirubin, and prothrombin time between the two groups were markedly diminished. Details are shown in Table 1.

**Table 1:** Baseline characteristics of patients in the heparin group and the control group.

	Before PSM				After PSM			
	Heparin	Control	*P* value	SMD	Heparin	Control	*P* value	SMD
	n = 818	n = 697			n = 818	n = 818		
Age, years	60.1 ± 16.0	62.2 ± 15.8	<0.001	−0.138	60.1 ± 16.0	61.6 ± 16.1	0.071	−0.093
Male, n (%)	491 (60.0)	433 (62.1)	0.404	–	491 (60.0)	499 (61.0)	0.686	–
CCI	4.26 ± 3.0	4.81 ± 2.9	0.013	−0.186	4.26 ± 3.0	4.4 ± 2.9	0.211	−0.047
Heart rate, beats/min	90.1 ± 17.8	88.8 ± 16.6	0.167	0.075	90.1 ± 17.8	90.0 ± 17.0	0.986	0.006
Temperature, °C	37.0 ± 0.8	36.9 ± 0.7	0.005	0.132	37.0 ± 0.8	37.0 ± 0.7	0.252	0.000
RR, breaths/min	20.1 ± 4.2	20.1 ± 4.2	0.984	0.000	20.1 ± 4.2	19.7 ± 4.0	0.039	0.097
MAP, mmHg	78.0 ± 10.9	77.7 ± 9.8	0.551	0.029	78.0 ± 10.9	78.0 ± 10.9	0.828	0.000
pH	7.35 ± 0.08	7.36 ± 0.08	0.049	−0.125	7.35 ± 0.08	7.37 ± 0.08	0.002	−0.250
PaO_2,_ mmHg	169.5 ± 76.0	168.2 ± 78.8	0.762	0.017	169.5 ± 76.0	176.7 ± 82.3	0.066	−0.091
PaCO_2,_ mmHg	39.7 ± 9.5	39.4 ± 8.9	0.627	0.033	39.7 ± 9.5	39.5 ± 9.1	0.752	0.021
Oxygenation index	276.3 ± 126.2	282.1 ± 124.1	0.366	−0.046	276.3 ± 126.2	290.2 ± 121.1	0.023	−0.112
Bicarbonate	21.8 ± 4.6	21.2 ± 4.5	0.024	0.132	21.8 ± 4.6	21.3 ± 4.5	0.044	0.110
Lactate, mmol/L	3.4 ± 2.8	3.5 ± 2.9	0.250	−0.035	3.4 ± 2.8	3.5 ± 2.9	0.478	−0.035
WBC count, 10^9/L	13.6 ± 8.2	13.45 ± 8.5	0.512	0.018	13.6 ± 8.2	12.9 ± 6.3	0.029	0.095
Lymphocyte count, 10^9/L	1.3 ± 3.7	1.3 ± 2.9	0.872	0.000	1.3 ± 3.7	1.2 ± 1.0	0.433	0.036
Hemoglobin, g/dL	9.9 ± 2.2	9.5 ± 2.3	<0.001	0.178	9.9 ± 2.2	9.6 ± 2.3	0.020	0.134
Platelet count, 10^9/L	205.7 ± 121.8	171.0 ± 115.1	<0.001	0.292	205.7 ± 121.8	196.0 ± 134.3	0.126	0.076
ALT, IU/L	202.0 ± 707.6	170.2 ± 629.1	0.359	0.047	202.0 ± 707.6	199.2 ± 820.4	0.940	0.004
Albumin, g/dL	2.9 ± 0.6	3.0 ± 0.6	0.004	−0.167	2.9 ± 0.6	3.0 ± 0.6	0.020	−0.167
Total bilirubin, mg/dL	1.8 ± 3.1	2.8 ± 5.3	<0.001	−0.235	1.8 ± 3.1	1.7 ± 3.3	0.874	0.031
Blood glucose, mg/dL	119.7 ± 40.0	122.1 ± 44.7	0.261	−0.057	119.7 ± 40.0	119.7 ± 43.0	0.984	0.000
Creatinine, mg/dL	1.6 ± 1.4	1.5 ± 1.2	0.131	0.076	1.6 ± 1.4	1.5 ± 1.2	0.105	0.076
Prothrombin time, s	16.3 ± 6.7	18.2 ± 8.8	<0.001	−0.246	16.3 ± 6.7	16.2 ± 6.6	0.751	0.015

CCI: Charlson Comorbidity Index, RR: Respiratory Rate, MAP: Mean Arterial Pressure, PaO_2_:Partial Pressure of Oxygen, PaCO_2_: Partial Pressure of Carbon Dioxide, WBC: White Blood Cell, ALT: Alanine Aminotransferase.

#### Prognosis of the heparin group and the control group.

**Fig 3:**
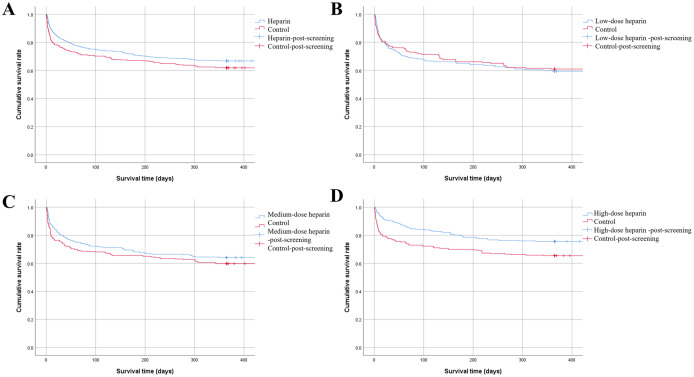
Survival analysis among different dosage heparin groups and the control group. **A**: Univariate Survival Curve: The one-year cumulative survival rate in the heparin treatment group was significantly higher than that in the control group (Log Rank = 6.024, P = 0.014); **B**: Univariate Survival Curve: There was no difference in the one-year cumulative survival rate between the low-dose heparin group and the control group (Log Rank = 0.075, P = 0.784); **C**: Univariate Survival Curve: The difference in the one-year cumulative survival rate between the medium-dose heparin group and the control group (Log Rank = 2.169, P = 0.141); **D**: Univariate Survival Curve: The high-dose heparin group demonstrated superior one-year cumulative survival compared to controls (Log Rank = 8.349, P = 0.004).

The short-term mortality in the heparin group (7-day mortality: 8.3% vs. 14.3%, 28-day mortality: 17.1% vs. 23.3%, 60-day mortality: 22.2% vs. 27.0%, all P-values < 0.05) was significantly lower than that in the control group. The one-year cumulative survival rate in the heparin group was significantly higher than that in the control group (Log Rank = 6.024, P = 0.014). However, patients in the heparin group had a longer ICU stay (6.6 ± 5.2 days vs. 5.6 ± 4.6 days, P < 0.001) and a longer duration of invasive mechanical ventilation (11.9 ± 6.8 hours/day vs. 9.3 ± 7.0 hours/day, P < 0.001) compared to the control group. Details can be found in Table 2 and Fig 3A.

**Table 2:** Prognosis of the heparin group and the control group.

	Heparin n = 818	Control n = 818	*P* value
Length of ICU stay, days	6.6 ± 5.2	5.6 ± 4.6	<0.001
Mortality			
7-day mortality, n (%)	68 (8.3)	117 (14.3)	<0.001
28-day mortality, n (%)	140 (17.1)	191 (23.3)	0.002
60-day mortality, n (%)	182 (22.2)	221(27.0)	0.025
Duration of respiratory support			
Invasive mechanical ventilation, h/d	11.9 ± 6.8	9.3 ± 7.0	<0.001
Non-invasive mechanical ventilation, h/d	0.1 ± 0.8	0.1 ± 0.8	0.639
Oxygen therapy, h/d	5.9 ± 5.8	5.2 ± 5.7	0.020
High-flow oxygen therapy, h/d	0.1 ± 0.7	0.5 ± 2.3	<0.001
No respiratory support, h/d	6.1 ± 5.5	9.0 ± 6.4	<0.001

#### The impact of different doses of heparin on the prognosis of patients with NPS-ARDS.

The aforementioned study indicates that heparin can improve the mortality of patients with NPS-ARDS. To further investigate the impact of different doses of heparin on patient prognosis, we divided the patients into different subgroups based on the dosage of heparin used. Using Propensity Score Matching (PSM) to match patients with similar baseline characteristics within different subgroups, we studied the effects of various doses of heparin on the prognosis of NPS-ARDS patients.

### Low-dose heparin group

#### Baseline characteristics of the low-dose heparin group and the control group (PSM).

After conducting PSM, the standardized mean differences (SMD) were significantly reduced. Both groups predominantly consisted of elderly males with similar Charlson Comorbidity Index scores (4.7 ± 3.1 vs. 4.6 ± 3.1, P = 0.819). The vital signs of both groups (heart rate, temperature, respiratory rate, and mean arterial pressure) were generally normal. Both groups exhibited mild compensatory metabolic acidosis, with higher PaO2 levels and PaCO2 within normal range. Both groups had elevated lactate levels (3.7 ± 3.6 mmol/L vs. 3.7 ± 3.0 mmol/L, P = 0.829), higher white blood cell counts (13.7 ± 8.0 * 10^9/L vs. 13.2 ± 6.4 * 10^9/L, P = 0.520), and prolonged prothrombin times (17.5 ± 7.8 s vs. 17.2 ± 7.6 s, P = 0.669). Details are shown in supplemental materials.

### The effect of low-dose heparin on the prognosis of patients with NPS-ARDS

There was no statistically significant difference in mortality and one-year cumulative survival rate between the two groups (P values were all greater than 0.05). Patients in the low-dose heparin treatment group had longer durations of invasive mechanical ventilation (11.5 ± 6.7 h/d vs. 8.5 ± 6.9 h/d, P < 0.001) and ICU stays (6.5 ± 4.3d vs. 5.4 ± 5.1d, P = 0.030) compared to the control group. Low-dose heparin treatment did not improve the prognosis of patients with NPS-ARDS. See Table 3 and Fig 3B for details.

**Table 3:** Prognosis of the low-dose heparin group and the control group.

	Low-dose Heparin	Control	*P* value
	n = 192	n = 192	
Length of ICU stay, days	6.5 ± 4.3	5.4 ± 5.1	0.030
Mortality			
7-day mortality, n (%)	19 (9.9)	26 (13.5)	0.267
28-day mortality, n (%)	45 (23.4)	42 (21.9)	0.715
60-day mortality, n (%)	57 (29.7)	46 (24.0)	0.205
Duration of respiratory support			
Invasive mechanical ventilation, h/d	11.5 ± 6.7	8.5 ± 6.9	<0.001
Non-invasive mechanical ventilation, h/d	0.1 ± 1.1	0.1 ± 0.9	0.922
Oxygen therapy, h/d	5.0 ± 5.6	6.4 ± 6.2	0.021
High-flow oxygen therapy, h/d	0.1 ± 0.7	0.3 ± 2.1	0.112
No respiratory support, h/d	7.3 ± 6.0	8.6 ± 5.6	0.029

### Medium-dose heparin group

#### Baseline characteristics of the medium-dose heparin group and the control group (PSM).

After performing propensity score matching (PSM), the standardized mean difference (SMD) for the two groups significantly decreased. Both groups consisted of elderly male patients, with the control group being slightly older (62.8 ± 15.6 years vs. 60.2 ± 16.2 years, P = 0.035). The vital signs of both groups (heart rate, temperature, respiratory rate, and mean arterial pressure) were generally normal and showed no statistically significant differences (all P values > 0.05). The lactate levels (3.4 ± 2.7 mmol/L vs. 3.5 ± 3.0 mmol/L, P = 0.701) and white blood cell counts (13.3 ± 7.9*10^9/L vs. 12.6 ± 6.0*10^9/L, P = 0.177) were relatively high in both groups. Details are shown in supplemental materials.

### The effect of medium-dose heparin on the prognosis of patients with NPS-ARDS

Compared with the control group, patients in the medium-dose heparin treatment group had lower 7-day mortality rates (10.6% vs. 14.7%), 28-day mortality rates (19.4% vs. 24.7%), and 60-day mortality rates (25% vs. 30.3%), but the differences were not statistically significant (all P values > 0.05). There was no statistically significant difference in the one-year cumulative survival rate between the two groups (Log Rank = 2.169, P = 0.141). Patients in the medium-dose heparin treatment group had longer ICU stays (6.6 ± 4.8d vs. 5.5 ± 4.3d, P = 0.002) and longer durations of invasive mechanical ventilation (12.3 ± 6.7h/d vs. 9.5 ± 7.1h/d, P < 0.001). Medium-dose heparin treatment did not improve the mortality of NPS-ARDS patients. Details can be found in Table 4 and Fig 3C.

**Table 4:** Prognosis of the medium -dose heparin group and the control group.

	Medium-dose Heparin	Control	*P* value
	n = 360	n = 360	
Length of ICU stay, days	6.6 ± 4.8	5.5 ± 4.3	0.002
Mortality			
7-day mortality, n (%)	38(10.6)	53(14.7)	0.093
28-day mortality, n (%)	70(19.4)	89(24.7)	0.088
60-day mortality, n (%)	90(25.0)	109(30.3)	1.113
Duration of respiratory support			
Invasive mechanical ventilation, h/d	12.3 ± 6.7	9.5 ± 7.1	<0.001
Non-invasive mechanical ventilation, h/d	0.1 ± 0.6	0.1 ± 0.7	0.505
Oxygen therapy, h/d	5.6 ± 5.5	4.9 ± 5.5	0.098
High-flow oxygen therapy, h/d	0.1 ± 0.9	0.5 ± 2.5	0.003
No respiratory support, h/d	6.1 ± 5.4	9.1 ± 6.7	<0.001

### High-dose heparin group

#### Baseline characteristics of the high-dose heparin group and the control group (PSM).

Similarly, after performing propensity score matching (PSM), the SMD for the two groups significantly decreased. Both groups predominantly consist of elderly males, and the Charlson comorbidity index is similar (3.8 ± 2.7 vs. 4.0 ± 2.7, P = 0.383). The respiratory rate is elevated within the normal range, while other vital signs are normal. The acid-base balance, PaCO₂, oxygenation index, and bicarbonate levels are normal, with PaO₂ being higher. Both groups had elevated lactate (3.0 ± 2.2 mmol/L vs. 3.2 ± 2.7 mmol/L, P = 0.235) and white blood cell counts (14.1 ± 8.8*10^9/L vs. 13.0 ± 6.6*10^9/L, P = 0.095). Details are shown in supplemental materials.

### The effect of high-dose heparin on the prognosis of patients with NPS-ARDS

The 7-day mortality (4.1% vs. 14.3%), 28-day mortality (9.4% vs. 22.6%), and 60-day mortality (13.2% vs. 24.8%) in the high-dose heparin group were all significantly lower than those in the control group, with differences being statistically significant (all P values < 0.05). The one-year cumulative survival rate was significantly higher in the high-dose heparin group compared to the control group (Log Rank = 8.349, P = 0.004). Patients in the high-dose heparin group had a longer ICU stay (6.7 ± 6.2d vs. 5.7 ± 4.8d, P = 0.041) and longer duration of invasive mechanical ventilation (11.7 ± 6.9 h/d vs. 5.7 ± 4.8 h/d, P < 0.001). High-dose heparin significantly improved the mortality of patients with NPS-ARDS. See Table 5 and Fig 3D for details.

**Table 5:** Prognosis of the high-dose heparin group and the control group.

	High -dose Heparin	Control	*P* value
	n = 266	n = 266	
Length of ICU stay, days	6.7 ± 6.2	5.7 ± 4.8	0.041
Mortality			
7-day mortality, n (%)	11(4.1)	38(14.3)	<0.001
28-day mortality, n (%)	25(9.4)	60(22.6)	<0.001
60-day mortality, n (%)	35(13.2)	66(24.8)	0.001
Duration of respiratory support			
Invasive mechanical ventilation, h/d	11.7 ± 6.9	5.7 ± 4.8	<0.001
Non-invasive mechanical ventilation, h/d	0.1 ± 0.7	0.1 ± 0.7	0.973
Oxygen therapy, h/d	6.9 ± 6.3	4.8 ± 5.4	<0.001
High-flow oxygen therapy, h/d	0.0 ± 0.2	0.5 ± 2.2	<0.001
No respiratory support, h/d	5.2 ± 4.9	9.2 ± 6.6	<0.001

## Discussion

Acute respiratory distress syndrome (ARDS) is caused by diffuse inflammation and edema in the lungs, characterized by severe hypoxemia. ARDS can result from various causes, including lung infections, sepsis, aspiration, trauma, pancreatitis, transfusion, and drowning, with sepsis being a major cause of ARDS [13].

The pathogenesis of ARDS is highly complex, involving local and/or systemic lung injury, inflammation, and the activation and dysregulation of coagulation [5]. Coagulation disorders are one of the important causes of the development of ARDS. The deposition of fibrin in the alveolar-capillary and platelet aggregation both contribute to the occurrence of ARDS [14]. ARDS patients with coagulation disorders have a higher 28-day mortality [15]. Coagulation disorders also play a significant role in the development and progression of sepsis [16], Septic patients are generally in a hypercoagulable state. This is characterized by reduced antithrombin levels, decreased thrombomodulin leading to inhibited protein C activation, disrupted thrombin function, defects in anticoagulant pathways, and impaired fibrinolysis [17].

Heparin is composed of repeating disaccharide units of uronic acid (L-iduronic acid or D-glucuronic acid) and N-acetyl-D-glucosamine. Heparin exerts its anticoagulant effect by binding to antithrombin through its unique pentasaccharide sequence, thereby inhibiting the activation of factor Xa and factor IIa in the coagulation cascade [18]. Heparin is widely used in clinical practice for anticoagulation in conditions such as venous thromboembolism, coronary artery disease, and extracorporeal circulation (blood purification). In recent years, research has increasingly focused on the various effects of heparin beyond anticoagulation [19]. The application of heparin in inhibiting inflammatory responses is a current research hotspot. Heparin exhibits broad anti-inflammatory and immunomodulatory effects. It can regulate the expression of cytokines and chemokines, inhibit the infiltration of immune cells, and downregulate levels of inflammatory factors such as IL-6 and TNF-α [20].

In addition to its role as an anticoagulant, heparin can improve outcomes in septic patients through various mechanisms [21]. During sepsis, heparin can bind to heparin-binding proteins, which helps mitigate the damage to the vascular endothelium caused by these proteins and reduces pulmonary edema [22]. Elevated histones can damage cell membranes, leading to significant calcium influx. Heparin can bind to histones, thereby mitigating the cell toxicity caused by these elevated histones [23]. Heparin can also reduce the production and release of inflammatory mediators during sepsis, including IL-6, TNF-α, and IL-8 [24]. Similarly, numerous studies have shown that heparin can improve outcomes in ARDS through various mechanisms. The destruction of the alveolar barrier leading to pulmonary edema is a key mechanism in the pathogenesis of ARDS [1]. Tight junctions between alveolar epithelial cells play a crucial role in maintaining the integrity of the alveolar barrier [25]. Heparin can mitigate the destruction of tight junctions between lung epithelial cells induced by endotoxins [26]. Fibrin deposition in the alveoli can lead to pulmonary shunting (ventilation-perfusion mismatch) and pulmonary fibrosis. Heparin can improve the ventilation-perfusion ratio in ARDS patients by reducing alveolar fibrin deposition and consequently decreasing shunting [27]. Heparin can also inhibit the growth of bacteria and viruses in the lungs by limiting their adhesion to the respiratory tract surface [28]. Research has also indicated that heparin can reduce the progression of lung injury in patients at risk for ARDS and shorten hospital stays [7]. This study used nebulized heparin. Compared with systemic administration of heparin (which was used in our study), nebulized heparin has the following characteristics: Nebulized heparin trials generally showed modest improvements in pulmonary physiology but no consistent mortality benefit. In contrast, our systemic high-dose subgroup demonstrated a significant survival advantage. Nebulized delivery is associated with minimal systemic absorption and thus lower bleeding risk, whereas systemic high-dose therapy raises concern for bleeding and HIT (though our study could not quantify these risks). Nebulized heparin may improve local pulmonary outcomes, systemic high-dose administration may be required to modulate the widespread coagulation and inflammatory derangements characteristic of NPS-ARDS. Basic research has also found that heparin can alleviate endotoxin-induced ARDS in mice by inhibiting the JAK2/STAT3 signaling pathway [29]. Aerosolized heparin can reduce fibrin, tissue factor, plasminogen activator inhibitor-1, plasminogen, and fibrin degradation products in the alveoli of mice induced by lipopolysaccharides, thereby alleviating lung injury in these mice [6].

Current research primarily focuses on the heparin for treating sepsis or ARDS, with relatively fewer studies exploring its preventive use in treating ARDS induced by sepsis. The Medical Information Mart for Intensive Care (MIMIC) is a large medical database established through a collaboration between Beth Israel Deaconess Medical Center (BIDMC) and the Massachusetts Institute of Technology (MIT). It provides clinical research data to researchers worldwide [10]. This study focuses on patients with NPS-ARDS from the MIMIC database, examining the impact of heparin use on their prognosis.

Compared with previous studies, our research demonstrates certain innovations. A study published in The Lancet Respiratory Medicine [7] investigated patients with ARDS (including both pulmonary and non-pulmonary etiologies), primarily evaluating the effect of nebulised heparin as an adjunctive therapy for lung injury. In contrast, our study provides novel insights in several important aspects. First, our study population was limited to patients with NPS-ARDS, a subgroup characterized by distinct inflammatory and coagulation features compared with pulmonary ARDS. To date, no prior research has specifically evaluated the effect of heparin in this patient population. Second, our study employed systemic subcutaneous administration of heparin, rather than nebulised administration. This approach reflects the most commonly used route in the ICU and does not require specialized delivery devices, making it more practical for routine clinical application. Third, a distinctive feature of our work was the dose-dependent analysis. By stratifying patients into low-, medium-, and high-dose groups according to daily heparin exposure, we demonstrated that only higher doses (>10,000 units/day) were associated with significant survival benefits, whereas lower doses showed no clear advantage. This dose–response relationship has not been previously reported. Finally, beyond short-term mortality, we also assessed the 1-year cumulative survival rate. This provides novel evidence supporting the potential long-term benefit of systemic heparin therapy in patients with NPS-ARDS.

The dosing groups for heparin usage were determined based on relevant literature and the distribution of samples in the database. When using heparin to treat ARDS, the usual administration method is local delivery through a vibrating mesh nebulizer, with doses ranging from 5000 IU/12h [30] to 25000 IU/6h [7]. Local heparin administration has minimal systemic effects on coagulation, allowing for relatively larger doses. When treating sepsis with heparin, it can be administered as a continuous intravenous infusion at 500 IU/h [31] or as a subcutaneous injection of 5000 IU/d [21]. Although the routes of heparin administration in these studies vary to some extent, they can provide reference for the heparin dose stratification in our research. In the MIMIC database, the heparin dosage distribution in patients with non-pulmonary sepsis-related ARDS is quite broad. Considering the use of integer nodes, these patients were grouped according to their heparin usage doses.

The results show that 1,515 patients were included in the study, of which 818 NPS-ARDS patients received heparin treatment. Subgroup analysis indicated that high-dose heparin (greater than 10,000 units/day) was required to achieve a reduction in mortality. Additionally, heparin use was associated with longer ICU stays and increased duration of invasive ventilation, which is consistent with the findings of Wang Deng [32]. While the > 10,000 U/day group showed benefit overall, future studies should more finely assess dose–response within the high-dose range, with careful attention to safety outcomes. Higher systemic doses of heparin may provide additional benefits by: More effectively suppressing thrombin generation and microvascular fibrin deposition. Enhancing anti-inflammatory effects, such as neutralizing circulating histones and modulating cytokine responses. Improving microcirculatory perfusion and limiting end-organ injury, which may be especially relevant in sepsis-driven ARDS. However, that these mechanistic explanations remain speculative. Prospective mechanistic studies are needed to validate whether the observed dose–response reflects true biological effects.

The prolonged ICU stay and ventilation time observed in the heparin group may be attributed to multiple factors. On one hand, bleeding complications not captured in the dataset may have extended ICU treatment, representing a major limitation of this study. On the other hand, patients receiving heparin—particularly those at higher baseline risk—may have experienced lower early mortality but required longer ICU recovery, leading to a “survivor effect” that passively prolonged ICU length of stay. In addition, our dose calculation method inherently ties drug exposure to ICU stay, which may introduce circular bias and exaggerate the association between the two.

The lack of benefit in the low- and medium-dose groups suggests that standard prophylactic doses may be insufficient to influence the systemic pathophysiological processes of NPS-ARDS. A threshold of >10,000 U/day may be required to achieve clinically meaningful effects. In addition to anticoagulation, heparin exerts dose-dependent anti-inflammatory and cytoprotective properties, such as neutralizing extracellular histones, inhibiting neutrophil extracellular trap formation, and mitigating endothelial injury. These effects may require higher systemic exposure. Although these mechanisms provide some biological rationale, we acknowledge that the observed benefit in the high-dose group may also reflect residual confounding factors. We emphasize the need for mechanistic and interventional studies to verify whether a true dose-dependent biological effect exists.

A major concern with heparin use is the potential for complications. The most significant complication of heparin overdose is bleeding, which can be severe and lead to intracranial hemorrhage. Additionally, heparin can cause heparin-induced thrombocytopenia (HIT), which can significantly impact patient outcomes [33]. Due to the limitations of the MIMIC-IV database, many bleeding events are heterogeneously or incompletely coded, and HIT diagnoses are rare and often lack confirmatory testing. Therefore, we were unable to accurately obtain detailed information on bleeding and HIT events attributable to heparin use. Although our findings suggest that higher-dose heparin confers a survival benefit, its safety profile remains uncertain. Until results from prospective trials with comprehensive safety reporting are available, the risk–benefit balance must be interpreted with caution. In previous research, our team developed a machine learning-based model to predict the occurrence of ARDS in patients with non-pulmonary infection sepsis. Administering heparin treatment to non-pulmonary sepsis patients at high risk of developing ARDS can reduce complications from excessive heparin use, achieving the goal of precision treatment.

This study is a single-center retrospective analysis, which inherently carries limitations such as selection bias and the inability to control for all potential confounding variables. Although PSM was applied to reduce confounding and improve comparability between groups, it cannot fully eliminate the influence of unmeasured or unknown confounders. The data for this study were extracted from the MIMIC-IV database. During data cleaning process, multiple imputation was applied to address missing values. Although samples with <95% data completeness were excluded, this approach may still introduce potential bias due to non-random patterns in residual missing data. The heparin dosing categorization (total ICU dose divided by ICU days) may oversimplify pharmacokinetic dynamics, as it does not account for administration frequency, dose adjustments during treatment. Future research will require multi-center prospective studies to validate our findings. Furthermore, relying solely on platelet count and PT cannot fully represent coagulation/bleeding risk profiles, particularly in the high-dose subgroup, which constitutes an important limitation of this study.

## Conclusion

High-dose heparin was associated with significantly improved short- and long-term survival, albeit at the cost of prolonged ICU stay and mechanical ventilation.

## Supporting information

S1 Table Anonymized clinical dataset used in the primary analyses. (XLSX)

S2 Text MIMIC database license. (PDF)

S3 File SQL script for data extraction. (SQL)

S4 File Python code used for data preprocessing, multiple imputation, and propensity score matching analyses. (PY)
