# Supplementary material for: Heparin improves the mortality of patients with non-pulmonary sepsis-associated ARDS: A MIMIC-IV database analysis based on propensity score matching
Source: PLoS One. 2025 Oct 10;20(10):e0333795. doi: 10.1371/journal.pone.0333795 (PMC12513587; doi:10.1371/journal.pone.0333795)
Supplement: S2 Text — (PDF) [file pone.0333795.s002.pdf]

CITI PROGRAM

Completion Date	13-Mar-2023
Expiration Date	13-Mar-2026
Record ID	48886665

This is to certify that:

Jinfeng Lin

Not valid for renewal of certification through CME.

Has completed the following CITI Program course:

Human Research
(Curriculum Group)
Data or Specimens Only Research
(Course Learner Group)
2 - Refresher Course
(Stage)

Under requirements set by:

Massachusetts Institute of Technology Affiliates

CITI
Collaborative Institutional Training Initiative

101 NE 3rd Avenue, Suite 320
Fort Lauderdale, FL 33301 US
www.citiprogram.org

Verify at www.citiprogram.org/verify/?w7f55584f-907f-4184-87dd-07c4afb1e9ac-48886665
